# Precision-guided therapy in dialysis-dependent classic hairy cell leukemia: a case report

**DOI:** 10.3389/fonc.2026.1895013

**Published:** 2026-08-31

**Authors:** Sindhusha Veeraballi, Madhu Bhargavi Chandra, Eric J. Vick

**Affiliations:** 1 Division of Hematology and Oncology, University of Cincinnati Cancer Center, Cincinnati, OH, United States; 2 Department of Internal Medicine, Wellstar Spalding Medical Center, Griffin, GA, United States

**Keywords:** cladribine, end stage renal disease (ESRD), hairy cell leukemia, obinutuzumab, vemurafenib

## Abstract

**Background:**

Classic hairy cell leukemia (HCL) is a rare, indolent B-cell lymphoproliferative disorder characterized by bone marrow fibrosis causing pancytopenia, splenomegaly, and a near-universal BRAF V600E mutation. Purine nucleoside analogs (PNAs) are the standard first-line therapy but are contraindicated in patients with significantly advanced chronic kidney disease (CKD) or end-stage renal disease (ESRD) due to renal excretion and risk of prolonged myelosuppression. Data on the use of targeted therapies, such as BRAF inhibitors, in dialysis-dependent HCL patients are lacking.

**Case:**

We present a novel case of a 43-year-old man on chronic hemodialysis secondary to suspected autosomal dominant polycystic kidney disease diagnosed with classic BRAF V600E-mutated HCL, treated with low-dose vemurafenib in combination with anti-CD20 therapy. The patient achieved hematologic remission without significant renal or infectious complications. Notably, this case is further distinguished by the development of reactive macrocytic polycythemia with normal erythropoietin levels and no alternative identifiable cause, highlighting an unusual hematologic manifestation in the setting of treated HCL and end-stage renal disease.

**Discussion:**

This report highlights the feasibility and efficacy of BRAF-targeted therapy combined with an anti-CD20 antibody in a young dialysis-dependent HCL patient, expanding therapeutic options for this high-risk population. Further prospective studies and case series are needed to guide management in this unique clinical context.

## Introduction

Hairy cell leukemia (HCL) is a rare, indolent B-cell lymphoproliferative disorder that accounts for less than 2% of all leukemias (1, 2). The median age of HCL diagnosis is 63 years in males (1). Classic HCL is characterized by pancytopenia, splenomegaly, and a near-universal BRAF V600E mutation. Nearly 90% of HCL cases harbor the BRAF V600E mutation, which drives constitutive activation of the MAP Kinase pathway and underpins the rationale for modern targeted therapies (1, 3, 4).

Bone marrow fibrosis, driven by cytokine-induced reticulin deposition, is a characteristic histopathologic feature of HCL and often results in a dry tap during bone marrow aspiration. About 10% of patients will also have a hypocellular marrow on biopsy, reflecting associated cytopenias (2).

First-line therapy for HCL is purine nucleoside analogs (PNAs), most commonly cladribine or pentostatin, which induce durable complete remissions in 80–90% of patients (2, 5). PNAs are often combined with an anti-CD20-based regimen (Rituximab) either as first line during the consolidation phase or in the setting of early relapse (within 12–18 months of initial treatment). Concurrent use of Rituximab and a purine analog has been shown to yield higher response rates than sequential administration (5). However, both purine analogs are primarily eliminated by renal excretion, and their removal by intermittent hemodialysis has not been reliably demonstrated, raising significant concerns in patients with severe renal impairment or end-stage renal disease (ESRD). Current guidelines recommend considering alternative therapies such as interferon-α or targeted agents in patients with significant renal dysfunction or contraindications to purine analogs (2).

HCL is exceedingly rare in the ESRD and dialysis population, and published experience with purine analogs in this setting is limited to isolated case reports, often requiring dose adjustments and close monitoring for toxicity (6–8). To our knowledge, no published reports have described the use of BRAF inhibitors in dialysis-dependent patients with HCL.

Here, we report a unique case of a 43-year old man with ESRD on chronic hemodialysis, diagnosed with classic BRAF V600E-mutated HCL, who was successfully treated with prolonged low-dose vemurafenib due to QTc prolongation in combination with anti-CD20 therapy. This case highlights the feasibility, safety, and efficacy of this regimen in a patient population with very limited therapeutic options and underscores the need for further studies on the use of targeted therapies in HCL patients with severe renal impairment.

## Case report

**Figure 1:**
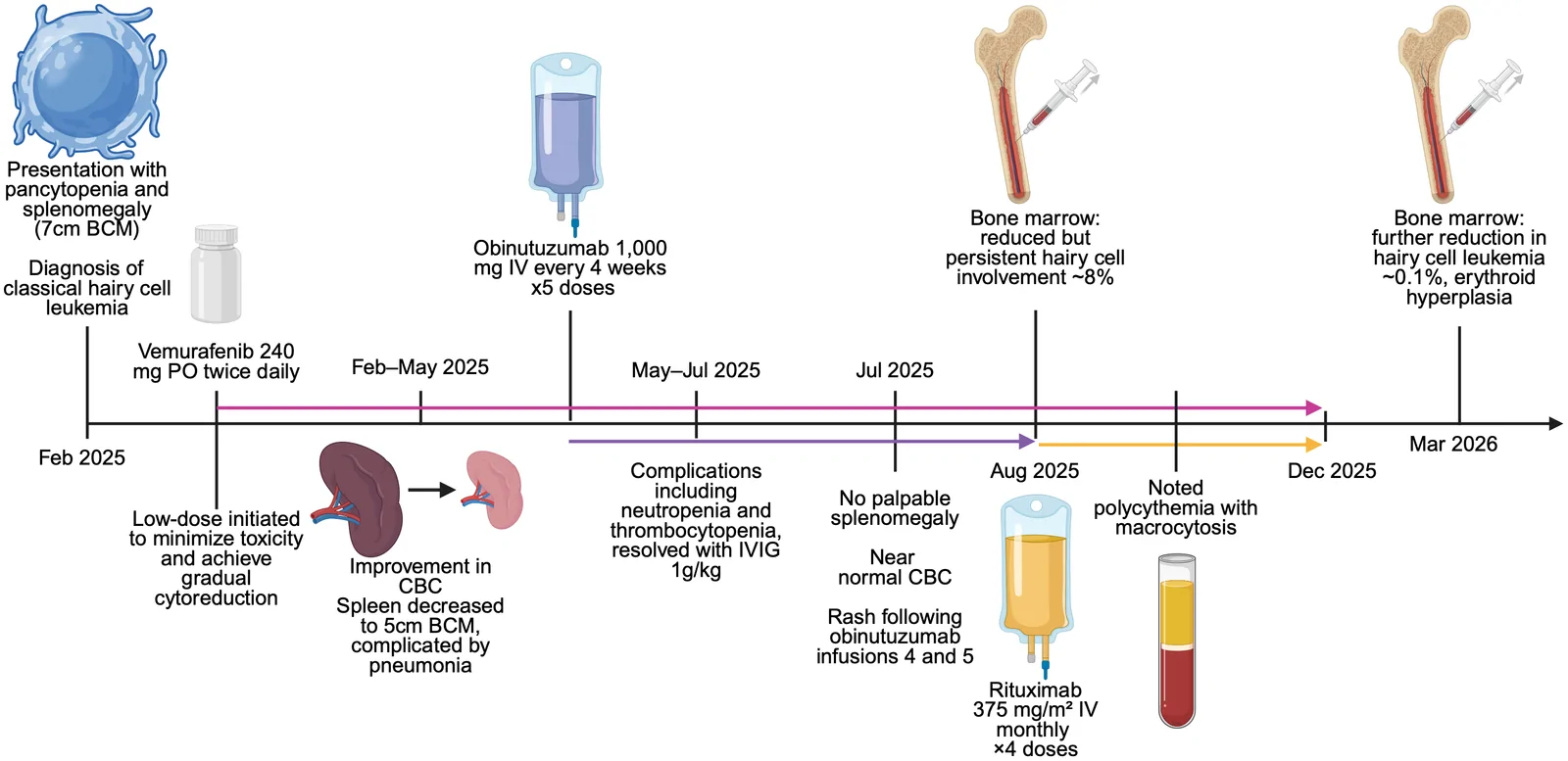
Treatment timeline and hematological response. Low dose vemurafenib was initiated in February 2025 which showed steady improvement in blood counts and reduced spleen size. After initial cytoreduction, obinutuzumab was added at a dose of 1,000 mg intravenously in May 2025 and continued till July 2025. Initial obinutuzumab doses were complicated by significant thrombocytopenia which responded to IVIG, and later doses complicated by rash. Bone marrow biopsy (BMBx) in august 2025 showed persistent disease. Obinutuzumab was replaced with Rituximab due to concerns for an allergic reaction while continuing low dose vemurafenib. Treatment stopped in December 2025 at the completion of therapy. BMBx in March 2026 showed significantly reduced hairy cell involvement.

A 43-year-old man with long-standing end-stage renal disease (ESRD) due to presumed autosomal dominant polycystic kidney disease was maintained on a three times weekly hemodialysis regimen since August 2018. His additional co-morbidities at the time of diagnosis included hypertension. He initially presented to an outside clinic for mild anemia (hemoglobin 11 g/dL) (reference range 13.5–17.5 g/dL) and thrombocytopenia (105×10^9^/L) in January 2024. His counts progressively declined, with Hb dropping to 6.4 g/dL and platelets to 32×10^9^/L by October 2024. However, no bone marrow biopsy or further hematological evaluation was pursued at that time. He presented to our clinic in January 2025 with progressive fatigue and easy bruising over the past 6 months. On physical examination, he had an extensive, non-petechial ecchymoses and a markedly enlarged spleen, palpable 7 cm below the left costal margin (**Figure 1**). No significant lymphadenopathy was noted. Vital signs were stable.

**Figure 2:**
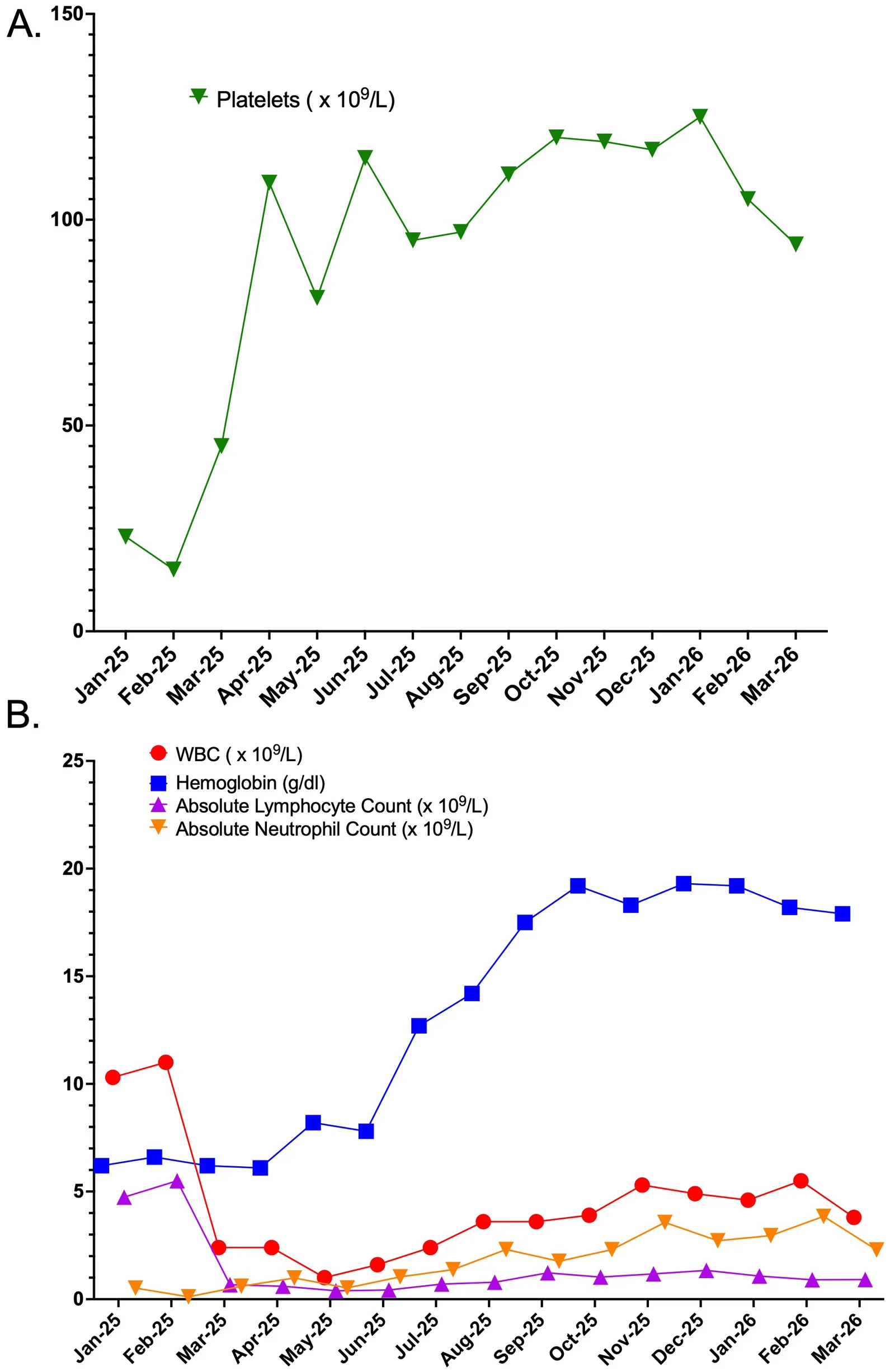
Hematologic parameters during the course of treatment. **(A)** Platelet count over the course of treatment. **(B)** White blood cell (WBC) count, hemoglobin, absolute lymphocyte count, and absolute neutrophil count over the course of treatment. Initial lymphocyte and WBC counts were elevated, reflecting circulating hairy cells identified on flow cytometry and peripheral blood smear. Following treatment initiation, the decrease in WBC and absolute lymphocyte count corresponded to the expected transient decline in neutrophils, followed by recovery of all hematologic parameters as bone marrow function improved. Hemoglobin continued to increase to the point of polycythemia and remained stable after completion of treatment.

Initial laboratory studies showed hemoglobin of 6.2 g/dL, hematocrit 24.2% (reference range 41–53%), and mean corpuscular volume (MCV) 91.2 fL (reference range 80–100 fL) (**Figure 2**). His white blood cell count was 10.3×10^9^/L (reference range 4.0–11.0×10^9^/L), with absolute lymphocytes 4,738/*μ*L (46%; reference range 1,000–4,800/*μ*L) and marked neutropenia with an absolute neutrophil count of approximately 0.515×10^9^/L (5%; reference range 1.5–7.5× 10^9^/L). Absolute monocytes and basophils were both reported as 0/*μ*L. Platelets were severely reduced at 23× 10^9^/L (reference range 150-400×10^9^/L). Atypical lymphocytes with morphology favoring hairy cells accounted for approximately 51% of the leukocytes on the peripheral smear, based on hematopathology review. There was no evidence of hemolysis (LDH, bilirubin, and haptoglobin all within normal limits). Renal function tests were consistent with known ESRD, with stable BUN and creatinine on dialysis.

Peripheral blood flow cytometry, performed on a pre-dialysis sample, demonstrated a monoclonal B-cell population expressing CD19, CD20, bright CD11c, and CD103, immunophenotypically consistent with classic HCL. A bone marrow biopsy in January 2025 showed a markedly hypercellular marrow with approximately 95% cellularity for age. The marrow was diffusely infiltrated by medium-sized lymphoid cells with oval or indented (“reniform”) nuclei, abundant pale cytoplasm, and irregular cytoplasmic projections morphologically consistent with hairy cells, with markedly reduced residual trilineage hematopoiesis. Immunohistochemistry demonstrated that the atypical B cells were positive for CD20, CD25, and Annexin A1. Molecular testing identified a BRAF V600E missense variant with a variant allele frequency of 75%. These findings established a diagnosis of classic HCL.

Given the patient’s ESRD on hemodialysis and the associated risk of drug accumulation and prolonged myelosuppression, standard first-line purine analog therapy with cladribine or pentostatin was considered high risk. After multidisciplinary discussion, including consultation with outside experts, a chemo-free targeted approach was chosen, personalized to the patient’s molecular diagnosis. Low-dose vemurafenib (240 mg orally twice daily) was initiated in February 2025 to minimize toxicity and achieve gradual cytoreduction. Attempts to increase the dose were limited by baseline QTc prolongation (510), necessitating continuation at this lower dose. Regular dermatologic examinations were performed during Vemurafenib therapy to monitor for secondary cutaneous malignancies associated with BRAF inhibitor therapy. From February through May 2025, his counts improved steadily: hemoglobin increased from approximately 6.2 g/dL at baseline to 7.5-7.9 g/dL by early May 2025, platelet count rose from about 23×10^9^/L to approximately 135-145×10^9^/L and the absolute neutrophil count increased from 0.5×10^9^/L to 2.6-3.3×10^9^/L over the same interval. He was hospitalized in April 2025 with influenza complicated by post-viral pneumonia, during which he missed four doses of vemurafenib; despite this brief interruption, hematologic parameters continued to improve, and spleen size decreased from approximately 7cm to 5cm below the left costal margin through May 2025, before obinutuzumab was introduced. After initial cytoreduction, anti-CD20 therapy with obinutuzumab was added at a dose of 1,000mg intravenously, administered weekly for 3 doses, followed by 2 additional cycles every 4 weeks through July 2025. The initial obinutuzumab doses were complicated by significant neutropenia and worsening thrombocytopenia, with platelet counts falling to approximately 10×10^9^/L; these cytopenias improved with one dose of intravenous immunoglobulin (1g/kg) and supportive care. Later doses (Doses 4 and 5) were complicated by a papular rash involving the chest and back, which resolved with steroids (**Figure 1**).

By July 2025, following several months of low-dose vemurafenib and five doses of obinutuzumab, the patient’s blood counts showed substantial improvement. His hemoglobin had risen to 15.9 g/dL, within the normal reference range, with an MCV of 106.4 fL (macrocytic). Platelets had improved to 101×10^9^/L, and the absolute neutrophil count was 2,826/*μ*L (approximately 2.83×10^9^/L) (**Figure 2**). On physical examination, the spleen was no longer palpable, indicating a marked reduction in splenomegaly. However, a follow-up bone marrow biopsy in August 2025 revealed persistent disease, with approximately 90% marrow cellularity and continued infiltration by atypical B lymphocytes. Immunohistochemistry remained positive for CD20, PAX5, BRAF, and Annexin A1, and negative for CD5 and CD10. Flow cytometry on the marrow aspirate demonstrated an abnormal clonal B-cell population comprising approximately 8.4% of total events, expressing CD103, bright CD11c, and CD123, consistent with residual, albeit significantly reduced, classic HCL. BRAF V600E was detected with a VAF of 8.9%. Given this incomplete response, the treatment regimen was continued, but anti-CD20 therapy was switched from obinutuzumab to rituximab because of the previous rash associated with obinutuzumab. Rituximab was administered at 375 mg/m^2^ intravenously once monthly for four doses, in combination with ongoing vemurafenib at 240 mg twice daily. The patient remained on thrice-weekly hemodialysis throughout. Vemurafenib was discontinued on infusion of dose four of rituximab.

Anemia improved significantly with HCL-directed treatment, which evolved into a progressive macrocytic erythrocytosis. Macrocytosis was first evident around June 2025, with an MCV of approximately 100.6 fL, which gradually increased to about 108 fL by October 2025 and remained persistently elevated thereafter. Hemoglobin, which had normalized with therapy, continued to rise, with erythrocytosis becoming apparent around September 2025 and reaching 20.2 g/dL by March 2026 (reference range 13.5–17.5 g/dL). He developed an erythematous, pruritic rash on his chest and extremities that was exacerbated by hot showers, and the rash persisted even after discontinuing vemurafenib. Despite his dialysis dependence, serum erythropoietin level was at the upper limit of normal for the testing laboratory (reference range 4–27 mIU/mL), an inappropriately high level in ESRD, where EPO is typically reduced. The patient had no exposure to exogenous EPO, no testosterone therapy, and no history of smoking or known chronic hypoxic lung disease. He had a functioning arteriovenous fistula, which could potentially contribute to shunt physiology and relative hypoxemia, but this alone was considered insufficient to explain the degree of erythrocytosis and would be unexpected in ESRD. Computed tomography of the abdomen and pelvis showed no renal or extrarenal mass lesions suggestive of ectopic EPO production. In view of the erythrocytosis, with worsening flow during dialysis and increased thrombotic risk, low-dose aspirin at 81 mg was started three times weekly with dialysis per nephrology preference.

A follow-up bone marrow examination in March 2026 showed persistent but significantly reduced hairy cell involvement. Conventional cytogenetic analysis demonstrated a normal male karyotype, 46, XY (20). Flow cytometry showed an abnormal lambda light chain–restricted B cell population representing 0.13% of total events, expressing CD19, CD20, CD103, CD11c, CD25, CD123, and HLA-DR, and negative for CD5, CD10, and CD34, consistent with minimal residual disease involving classic HCL. Next-generation sequencing no longer detected the previously identified BRAF V600E mutation and did not reveal any pathogenic variants in JAK2, CALR, or MPL.

Given the normalization of splenic size, improvement in peripheral blood counts, and the development of polycythemia, we elected to withhold further HCL-directed therapy at this time and continue close monitoring. Future treatment will be reconsidered if hematologic parameters again meet established indications for therapy, if splenomegaly recurs, or if new disease-related symptoms emerge. Planned treatment regimens would involve either a Bruton’s tyrosine kinase (BTK) inhibitor or venetoclax, which are both dialyzable.

## Discussion

HCL is a rare, chronic mature B-cell lymphoproliferative disorder characterized by circulating atypical lymphocytes, pancytopenia, marked monocytopenia, and splenomegaly (9). Classic HCL is diagnosed by the presence of “hairy” cells in the peripheral blood (PB) and/or bone marrow (BM), showing pale cytoplasm with fine, hairlike projections, together with a characteristic immunophenotype that typically includes expression of CD11c, CD25, CD103, CD123, CD20, CD22, CD200, DBA44 (CD72), tartrate-resistant acid phosphatase (TRAP), and annexin A1 (ANXA1). The presence of the somatic *BRAFV*600*E* mutation in the leukemic cells, detected in most classic HCL cases, further supports the diagnosis and helps distinguish it from HCL variant and other splenic B-cell neoplasms, and is necessary for disease pathogenesis (2).

Patients with HCL usually present with fatigue, constitutional symptoms, cytopenia-related manifestations (such as infections or bleeding), and splenomegaly (10). Treatment is not required at diagnosis in all patients, but is recommended when clinical symptoms or progressive hematologic deterioration occurs. In current practice, indications for therapy generally include the presence of at least one of the following: hemoglobin <11g/dL, platelet count <100,000/*μ*L, or absolute neutrophil count <1,000/*μ*L; symptomatic or progressive organomegaly; constitutional symptoms; or recurrent or severe infections. In our case, the patient met treatment criteria based on worsening cytopenias and splenomegaly, which prompted initiation of systemic therapy (2, 11).

Before the advent of purine nucleoside analogs (PNAs), the management of hairy cell leukemia (HCL) relied primarily on splenectomy and interferon-α, which typically produced only partial and often transient responses; many patients ultimately experienced progressive disease or succumbed to infectious complications (1). The introduction of PNAs such as cladribine and pentostatin in the late 1980s and early 1990s represented a major therapeutic breakthrough, leading to high complete remission rates, durable disease control, and a near-normal life expectancy for the majority of patients with classic HCL (1, 2, 12). Nevertheless, repeated PNA exposure in the relapsed setting has been associated with cumulative immunosuppression, an increased risk of severe infections and secondary malignancies, and the eventual development of resistance in a subset of patients after multiple treatment courses (1, 12–14).

In parallel, recent advances in the molecular characterization of HCL have substantially improved understanding of its pathogenesis. The discovery of the recurrent *BRAFV*600*E* mutation in over 90% of cases of classic HCL has established this lesion as a central oncogenic driver and a defining molecular hallmark of the disease, with important diagnostic and therapeutic implications (1, 3). The *BRAFV*600*E* mutation results in constitutive activation of the *BRAF* kinase and persistent signaling through the RAF–MEK–ERK pathway, driving aberrant transcriptional programs that promote survival and proliferation of leukemic cells. This lesion is now regarded as the molecular hallmark of classic hairy cell leukemia, with both diagnostic value and clear implications for targeted therapy using *BRAF* inhibitors (BRAFi), with or without concomitant MEK inhibition (3, 4). *In vitro* studies have shown that exposure of primary HCL cells to BRAFi and MEKi leads to rapid dephosphorylation of MEK/ERK, suppression of the RAS–RAF–MEK–ERK transcriptional output, loss of the HCL-specific gene expression signature, morphologic reversion of “hairy” cells toward a more normal appearance, and induction of apoptosis (15, 16).

Building on these molecular insights, several novel therapeutic options have emerged for classic HCL. BRAF inhibitors (vemurafenib, dabrafenib) (17, 18), either alone or in combination with MEK inhibitors (trametinib, binimetinib) (19), or anti-CD20 monoclonal antibodies (rituximab, obinutuzumab) (20, 21). Other targeted agents, including Bruton’s tyrosine kinase inhibitors (ibrutinib, zanubrutinib) (22, 23) and the anti-CD22 immunotoxin moxetumomab pasudotox, have also demonstrated clinical activity in HCL; however, the latter is no longer available in the United States (24). Venetoclax also represents a viable therapy which has shown efficacy in hairy cell leukemia and would be compatible with dialysis. Despite the discovery of novel targeted therapies, the treatment of relapsed/refractory HCL still remains a challenge. Although standard pharmacotherapy eliminates the bulk of the disease, some leukemic clones are protected in the bone marrow microenvironment by evading the innate immune system, with complex, incompletely understood underlying molecular mechanisms. Recent studies indicate that CXCR4 and its ligand, CXCL12, are key components of immune crosstalk between hairy cells and the leukemic microenvironment, and overexpression of CXCR4 is directly associated with disease progression, leukemic cell survival, and chemoresistance, making CXCR4 antagonists (e.g., plerixafor, BPRCX807) a future avenue to overcome resistance to current therapies (25, 26).

The introduction of BRAFi has significantly transformed the management of relapsed or refractory HCL. Vemurafenib, a selective B*RAFV*600*E* inhibitor, has produced hematologic response rates of approximately 95–100%, with complete remission (CR) rates of 30–45% when used as monotherapy, although remissions may be shorter and minimal residual disease (MRD) often persists. Dietrich et al. reported a 40% CR rate with low-dose vemurafenib (240–480 mg daily), with all patients experiencing rapid hematologic improvement (17). Falini et al. provide a comprehensive review of BRAF inhibitor strategies in relapsed/refractory HCL, highlighting their clinical benefit and safety profiles (12). More recently, combination regimens using BRAF and MEK inhibitors, such as dabrafenib plus trametinib, have shown high efficacy in BRAF V600E-mutated HCL, including in heavily pretreated patients (19). Combining BRAF inhibition with anti-CD20 monoclonal antibodies (rituximab or obinutuzumab) has produced even higher CR rates exceeding 85–90%, with frequent achievement of minimal residual disease (MRD) negativity (20, 21). Park et al. demonstrated that upfront vemurafenib plus obinutuzumab in newly diagnosed HCL achieved CR rates above 90% and MRD negativity in nearly all responders, supporting the use of this combination for deeper and more durable remissions (21).

In case of renal insufficiency, cladribine is contraindicated if creatinine clearance (CrCl) is ≤50 mL/min, and pentostatin is contraindicated if CrCl is <60 mL/min, due to limited data and a potential risk of increased toxicity, as PNAs and their metabolites are mainly excreted renally (1). There is no specific data on their use in patients with HCL and hemodialysis-dependent ESRD.

Pharmacokinetic data support the feasibility of using vemurafenib and obinutuzumab in ESRD. Vemurafenib is highly protein-bound and undergoes hepatic metabolism, with less than 1% of the administered dose excreted unchanged in the urine. Consequently, clinically relevant accumulation due to reduced glomerular filtration is unlikely, and renal dysfunction is not expected to have a major impact on overall drug exposure (27). Although formal pharmacokinetic studies in dialysis dependent patients are lacking, limited experience can be extrapolated from isolated case reports of metastatic melanoma in ESRD, including patients on hemodialysis, in whom vemurafenib was administered without unexpected toxicity. In one such report, a patient with metastatic melanoma and ESRD treated with vemurafenib achieved a significant tumor response and generally tolerated therapy well; the main adverse event was a grade 3 prolongation of the QTc interval after approximately five months of treatment, which was managed with dose reduction rather than discontinuation (28).

Obinutuzumab, a glycoengineered anti-CD20 monoclonal antibody, is catabolized via the reticuloendothelial system and does not rely on renal excretion or get affected by dialysis. Redfield et al. (29) confirmed that the pharmacokinetics and safety of obinutuzumab were comparable in patients with ESRD and those with normal renal function (28). HCL clinical trials on Vemurafenib and Obinutuzumab (eg, Park et al.) (21) typically excluded patients with severe CKD (creatinine >1.5×ULN). Thus, both agents are theoretically suitable for patients with advanced renal dysfunction, although formal pharmacokinetic and safety studies in dialysis-dependent patients are lacking.

A review of the literature reveals only isolated cases of HCL in patients with ESRD or kidney transplants (**Table 1**). In these cases, purine analogs were used with modified schedules and close monitoring, achieving remission without significant renal complications (6–8). Our report adds to this limited literature by demonstrating the feasibility of BRAF-targeted therapy in the dialysis-dependent population.

**Table 1:** Published cases of hairy cell leukemia in patients with end-stage renal disease or kidney transplant.

Author (year)	Age/sex	HCL subtype	Therapy	Renal Status	Outcome
Vinante et al (6). (2013)	51/F	Classic HCL	2-CDA (2-Chlorodeoxyadenosine, leustatin, cladribine) 0.1 mg/Kg IV once per week for 6 cycles	Kidney transplant (calcineurin immunosuppression)	Complete remission; graft maintained
Rankovic et al. (7) (2020)	75/M	Classic HCL	Cladrabine 0.14 mg/kg x 5 doses and rituximab 375 mg/m2 biweekly x 6 doses	ESRD on hemodialysis	Complete remission
Gozzetti et al. (8) (2023)	68/M	Classic HCL	Interferon-α followed by cladribine every other week	ESRD	Complete hematological remission and improvement of renal function

Another complication of our case was the evolution of erythrocytosis and polycythemia during treatment in the absence of identifiable secondary causes in ESRD. Bone marrow examination was only suggestive of classic hairy cell leukemia (HCL), without morphologic or immunophenotypic evidence of a concurrent myeloproliferative neoplasm or myelodysplasia, and EPO was within the normal range despite end−stage renal disease, arguing against both reactive and primary polycythemia.

Taken together, the coexistence of macrocytosis and polycythemia in this setting may reflect an HCL related perturbation of marrow architecture and erythropoiesis overcompensating rather than an independent clonal myeloproliferative process. One possible explanation is that the combination of marrow infiltration by HCL and subsequent therapeutic cytoreduction produced a state of dysregulated but effective erythropoiesis, manifesting as both macrocytosis and elevated red cell mass. Similar coexistence of HCL with true polycythemia vera has been described (30, 31), but in those reports, the erythrocytosis is attributable to a separate *JAK*2 mutated myeloproliferative neoplasm rather than to HCL itself, highlighting the novelty of our patient’s macrocytic erythrocytosis in the absence of a demonstrable myeloproliferative clone. Another proposed mechanism is BRAFi-induced paradoxical activation of downstream MAPK signaling in wild-type BRAF cells, as well as temporary delay of erythroid differentiation (32).

## Conclusion

In summary, purine nucleoside analogs remain the standard of care for classic HCL but pose substantial challenges in patients with end−stage renal disease because of their predominantly renal clearance, limited capacity for removal through dialysate, and profound, prolonged myelosuppression. In contrast, *BRAF* inhibitors, in combination with anti−CD20 monoclonal antibodies, provide a mechanistically distinct, highly active, and pharmacologically attractive alternative for patients with severe renal impairment. Our case demonstrates the feasibility of low−dose vemurafenib plus obinutuzumab in a dialysis−dependent patient with HCL, achieving hematologic remission with no major complications.

## Data Availability

The original contributions presented in the study are included in the article/supplementary material. Further inquiries can be directed to the corresponding author.
